# Perceived Conjunctival Foreign Material Egress in Morgellons Disease: A Case Study

**DOI:** 10.1155/2024/9952722

**Published:** 2024-05-10

**Authors:** Sean Ghiam, Badal Sojitra, Collin Reiff, Connie M. Sears, Justin N. Karlin

**Affiliations:** ^1^Sackler School of Medicine, New York State/American Program of Tel Aviv University, Tel Aviv, Israel; ^2^Department of Ophthalmology, Rutgers Robert Wood Johnson Medical School, New Brunswick, NJ, USA; ^3^Department of Psychiatry, Division of Addiction Psychiatry, New York University Langone Hospital, New York City, USA; ^4^Division of Orbital and Ophthalmic Plastic Surgery, Doheny and Stein Eye Institutes, University of California, Los Angeles, USA

## Abstract

The purpose of this report is to present a case of a 63-year-old man with orbital Morgellons disease. A 63-year-old man presented reporting 15 years of daily egress of different foreign bodies apparently found in the superior fornices of both eyes, exhibiting a classic manifestation known as the matchbox sign. He described the symptoms starting after a facial trauma. The patient stated that at several points over the 15-year course of his condition, he was so distressed that he had contemplated suicide. On multiple exams by a range of ophthalmic professionals, there was no evidence of foreign body. Further investigation involving MRI and plain radiographs demonstrated similar lack of findings. A trial of gabapentin was performed without improvement in symptoms. He discontinued care 5 months later. Morgellons disease is a poorly understood condition, particularly ophthalmic presentations of the disease. Despite extensive investigation, the exact cause of Morgellons disease remains unclear, and there is no definitive treatment for the condition. We highlight the importance of empathetic listening in building trust, as a means of helping the patient to seek psychological help.

## 1. Introduction

Morgellons disease (MD) is a specific manifestation of delusional disorder in which patients report the presence of fibers or other unusual substances within or extruding from the skin [1]. Among sufferers, the description of these fibers (e.g., color, texture, and caliber) varies greatly, and the perceived presence of such fibers may be accompanied by symptoms, such as itching, burning, or foreign body sensation [2–4]. While the etiology of MD is not well understood, it has been associated with a number of underlying conditions, including dermatologic diseases [1–5], autoimmune disorders [2], neuropsychiatric conditions [6, 7], and infections [8–10]. Any accompanying dermatologic lesions that are observed on examination may be believed to be self-inflicted, as these patients commonly attempt to eliminate the substance by using fingernails, tweezers, nail clippers, needles, toothpicks, or any other manner of instrument, homemade, or otherwise [1–4]. Therefore, a comprehensive physical examination with a dermatologist may be necessary to differentiate between primary lesions and excoriations.

There are no universally accepted diagnostic criteria for MD, and the diagnosis is often based on clinical observation and ruling out other potential causes of the symptoms [1]. Healthcare providers may conduct various tests to rule out infectious, dermatological, or psychiatric conditions that could explain the symptoms [3–6]. According to the American Psychiatric Association's Diagnostic and Statistical Manual of Mental Disorders (DSM)-V, the closest diagnosis is somatic type delusional disorder. This disorder is defined as the presence of one or more delusions lasting for 1 month or longer, with criteria for schizophrenia never having been met, functioning not markedly impaired, symptoms not due to another medical condition or substance, and not better explained by another mental disorder [1].

MD is not limited to being a skin condition, as there are several case reports that have documented MD involving the otic, nasal [11], and oral cavity [12]. Moreover, these patients are known to self-medicate with topical remedies like hydrogen peroxide, bleach, rubbing alcohol, or ointments [1–4]. Fatigue, headache, arthralgias, visual changes, gastrointestinal symptoms, mental confusion, and alterations in skin texture or pigmentation are among the more common generalized symptoms associated with MD [1–5].

While MD is a relatively rare condition, it has received significant attention in recent years due to the growing number of reports from patients who have experienced symptoms [13]. One study estimated that the prevalence of MD is 3.65 per 100,000 people [13]. However, the prevalence of the disease is difficult to determine, as it is a condition that is likely underdiagnosed and not well-studied [2].

We have identified two prior cases of periocular Morgellons [14, 15]. The diagnosis of periocular MD may be challenging, as many of the symptoms reported by patients can also be seen in other conditions, such as dry eye syndrome, periocular dermatitis, or blepharitis. However, recent studies [1–3] have suggested that a subset of patients with MD may have a unique underlying condition that requires further investigation and characterization.

Treatment for this condition is elusive, as there is currently no specific treatment for MD. It is best conceptualized as a delusional disorder, a rare psychotic illness characterized by delusions with contents that are theoretically possible but highly unlikely. Accordingly, the diagnosis is one of exclusion and should only be made after a thorough history, eye exam and work up are completed. Management of the condition often involves addressing the underlying psychological cause and providing symptomatic relief for the patient [3, 4]. In some cases, this involves the use of topical or systemic medications [16–20] in combination with psychotherapy [1, 20].

The aim of this study is to present a rare presentation of MD affecting the conjunctival fornices, hypothesizing that despite its controversial nature, MD can manifest beyond the skin. This unique case contributes to the existing literature by expanding the understanding of MD's clinical spectrum and raising awareness among clinicians about its potential variability in presentation. These findings underscore the importance of considering MD in the differential diagnosis of unexplained ocular symptoms, advocating for multidisciplinary approach to diagnosis and management. The report emphasizes the need for further research to elucidate the pathophysiology and optimal management strategies for MD, ultimately aiming to provide better care for affected individuals.

## 2. Case Presentation

A 63-year-old man presented to a tertiary care oculoplastic surgery practice endorsing a 15-year history of various foreign bodies emanating from the superior fornices of both eyes. The egress of these foreign bodies was associated with blurred vision, burning sensation, dryness, redness, and eye pain.

He states his symptoms began after colliding with a “trumpet” plant in 2004. He states that he “had both eyes wide open, and the flowers were in full bloom, replete with live insects and twig branches.” He states that flowers of the plant entered his mouth and eyes. He then “flushed out larger pieces of the plant and insect material from his eyes with an ‘eye cup' and a 4 oz saline wash.” Despite the efforts to wash out the debris, he continued to experience foreign body sensation, concluding that the persistence of symptoms is due to the presence of material in a “permanent laceration behind the ocular globe.” He reports insomnia and malaise in the days following the injury, accompanied by itching, eye pain, and pressure sensation. At some point during the days following this injury, he states that in a state of delirium, he found himself rubbing his face on the floor in an effort to relieve the itching sensation. He believes this was instrumental in making the injury worse.

**Figure 1:**
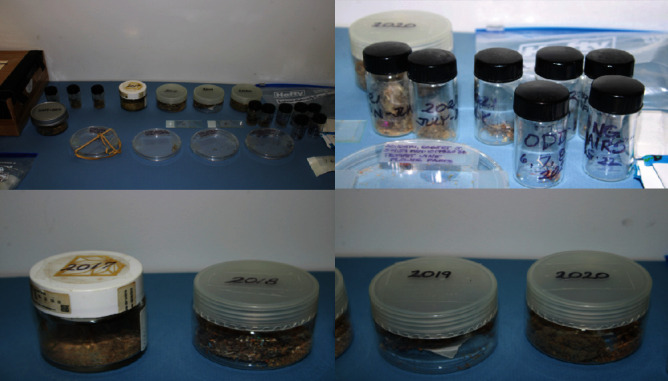
Foreign bodies collected in Petri dishes, vials, and various containers by patient between 2004 and 2023.

**Figure 2:**
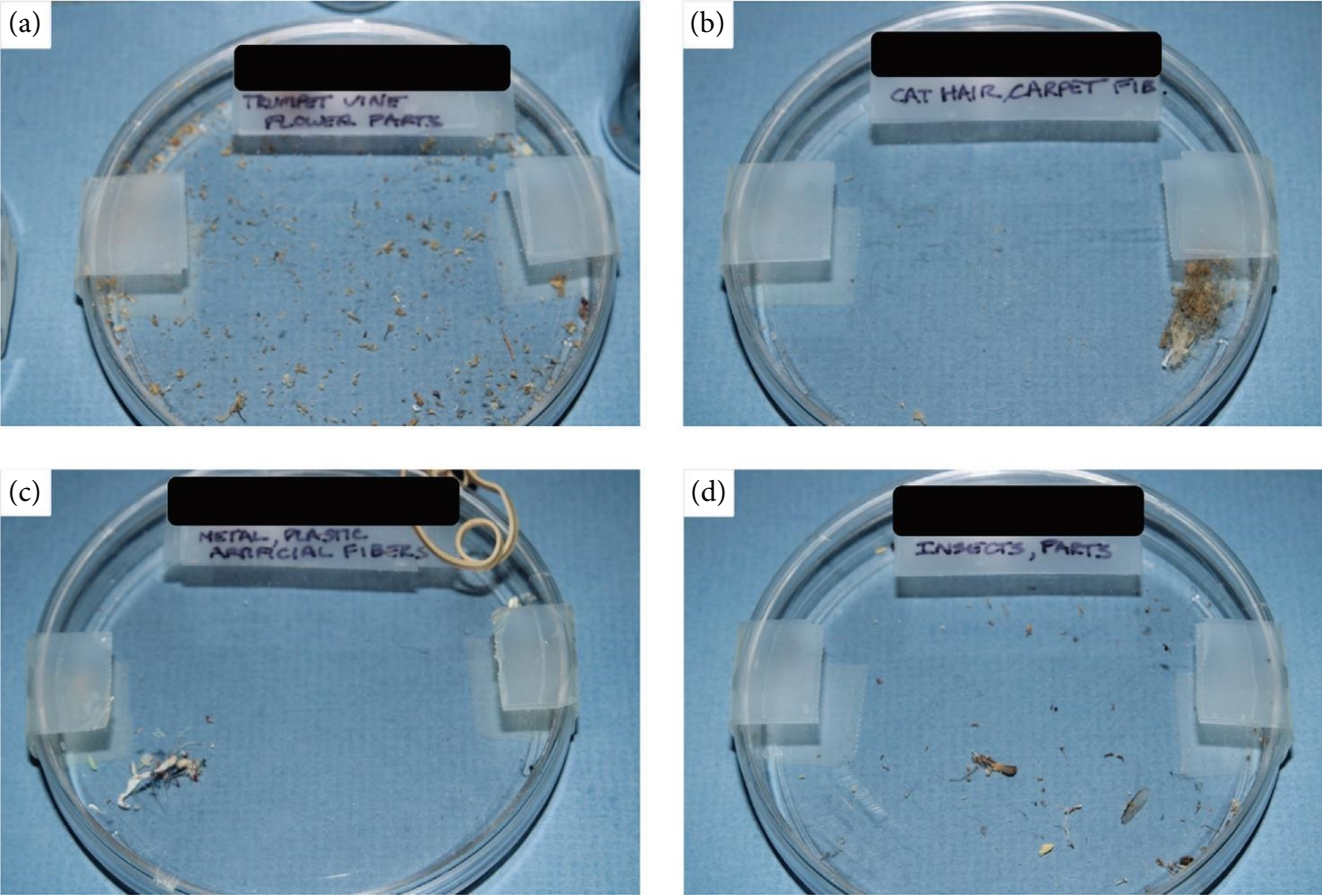
The patient provided samples of debris such as trumpet vine flower parts (a), acrylic carpet fibers, cat hair (b), thread, metal filaments (c), and insects (d) in Petri dishes to multiple ophthalmologists that he reports having collected from both eyes via daily eye flushing and using a mascara brush.

Over the ensuing years, the patient amassed a large collection of foreign bodies that he had purportedly extricated from his superior fornices (Figure 1). On presentation, the patient provided samples of debris, among these were trumpet vine flower parts (Figure 2(a)), acrylic carpet fibers, cat hair (Figure 2(b)), thread, metal filaments (Figure 2(c)), and insects (Figure 2(d)). He described a carefully choreographed ritual by which he collected this debris. He said, after meticulous cleaning of the kitchen sink, he would place a stopper over the drain, run the tap, and force water under the eyelids until the sink had filled completely. He would then use a “mascara brush” to collect any flotsam. This was followed by a slow draining of the sink water and collection of any sedimented debris.

He reported a prior history of presenting to multiple ophthalmologists and oculoplastic surgeons. Review of prior records uncovered multiple examinations by a number of board-certified ophthalmologists. These examinations repeatedly revealed no evidence of foreign bodies, including with eversion of lids and sweeping of fornices. An orbital plain radiograph obtained in March 2019 was within normal limits.

The patient reported that ongoing symptoms affected his ability to work. And the patient stated that many times in the years since the start of his condition, he had felt dejected and forsaken by the medical community. The repeated dismissal of his symptoms, in spite of communicating his continued distress, led the patient to experience recurrent suicidal ideation.

His past medical history was significant for coronary artery disease, chronic bronchitis, hypertension, hypercholesterolemia, and benign prostatic hyperplasia. Past surgical history included inguinal hernia repair and heart surgery. Past ocular history included a diagnosis of floppy eyelid syndrome in 2017. Family history was noncontributory. The patient denied any recent use of illicit drugs, alcohol, prescription narcotics, antidepressants, and had no history of prior mental illness or psychiatric counseling. Medications included aspirin, atenolol, finasteride, and atorvastatin. The patient reported a drug allergy to cephalexin (hives). Review of systems was positive for constitutional weight loss, sinus congestion, depression, back pain, joint/muscle pain stiffness, but negative for dermatologic lesions.

Ophthalmic examination in May 2019 revealed visual acuity with correction was 20/20 OD and 20/30 OS, with pinhole correction 20/20. External exam revealed floppy eyelid syndrome, Hertel exophthalmometry measured 17 mm OD and 15 mm OS at base 105. Pupils were equal, round, and reactive to light and accommodation. Motility examination demonstrated full ductions and versions. Slit lamp exam demonstrated trace conjunctival injection with corneal punctate staining bilaterally. Iris was unremarkable and lenses were clear bilaterally. Otherwise, no other ophthalmic masses, lesions, or ulcers were observed.

Further investigation in June 2019 with an MRI of the orbits demonstrated no abnormal findings. He was prescribed gabapentin and instructed to follow-up in 2 months. Upon return, he had no improvement in symptoms, was advised to refrain from flushing and touching eyes, and instructed to follow-up with a psychiatrist. He discontinued care 5 months later due to frustration with the pace of investigation and due to a perceived dismissal of his condition by labeling it as a psychiatric condition.

On presentation to our clinic in February 2023, he stated that his symptoms had somewhat resolved. He noted that the volume of material emanating in the fornices had decreased, and that there was noticeable diminishment in his ocular symptoms from 2019 to present. During the examination, the patient appeared well-groomed and cooperative, with fluent speech and appropriate demeanor. He was fully oriented and demonstrated intact memory, abstract thinking, insight, and judgment. He denied suicidal ideation but was concerned about the etiology of ophthalmic symptoms. Thorough examination of the eye was performed, including examination of the eye and fornix with eyelid eversion, and no lesions or foreign bodies were noted. The patient was allowed to explain his condition at length without interruption and was met with empathic listening to aid development of a therapeutic alliance. Although no psychotropic drugs were prescribed at the time, cognitive–behavioral therapy (CBT) and supportive psychotherapy were recommended to the patient given the traumatic nature of the experience and significant and persistent effects on his quality of life.

## 3. Discussion

The diagnosis and treatment of MD remains controversial due to the lack of clear diagnostic criteria and the absence of a specific medical treatment for the condition [1]. Many medical professionals consider the symptoms of MD to be psychosomatic, a subset of delusional parasitosis [3–6, 20], while others argue that MD may be a dermatological medical condition with an infectious etiology [8–10, 20]. Some researchers have proposed diagnostic criteria based on the presence of characteristic symptoms, such as the sensation of crawling or biting, the appearance of fibers or other materials in skin lesions, and the development of psychiatric symptoms [1, 20]. However, these criteria have not been widely accepted, and more research is needed to establish a reliable and valid diagnostic approach for MD. This has led to considerable variation in the reported prevalence [13] and clinical features of MD across different studies [10–15].

In addition to MD, the differential diagnosis for ophthalmic symptoms and sensations of foreign bodies in the eye may include conditions such as dry eye syndrome, blepharitis, conjunctivitis, corneal abrasions, and psychiatric disorders such as delusional parasitosis, which may present with similar symptoms but require distinct approaches to management. Diagnosis of ophthalmic, periocular, or orbital Morgellons is more challenging still due to the dearth of literature on such presentations of the disease. To our knowledge, only two previous accounts of MD have been reported in the ophthalmology literature. Both of these accounts involve lesions which were notable upon examination: one presenting with corneal perforation [14] and another presenting with an ulcerated upper eyelid lesion [15]. In contrast, our patient presented without any identifiable lesions, despite thorough ophthalmic and orbital examination, similar to other reports [11, 12]. This absence of visible lesions complicates the diagnosis and underscores the need for further research and understanding of MD, particularly in its less common presentations involving the eyes and periocular region.

It is tempting to speculate about whether or not the patient's initial injury somehow played a role in triggering the delusional disorder. The patient stated that the condition started after he “collided with a trumpet plant.” Angel's trumpet is the common name of a group of plants common to the American West. These flowering plants are members of Solanaceae, the nightshade family. *Brugmansia* spp. and *Datura* spp. are known to contain a variety of tropane alkaloids, including atropine, scopolamine, and hyoscyamine, substances with psychoactive and anticholinergic properties [21]. Psychotic episodes have been reported with ingestion of even small amounts of plant material from the aforementioned species [22, 23]. It is well-known that patients with MD often have psychiatric comorbidities [6, 7], and it is feasible that the patient described herein experienced a psychotic spectrum disorder, most likely a delusional disorder, after being exposed to the aforementioned alkaloids.

As with delusional disorders in general, the treatment of MD remains a challenge, and there is no single approach that has been proven to be effective for all patients. It should be noted that any attempt to present evidence that contravenes the patient's delusion will risk catalyzing the patient's hostility and damaging therapeutic alliance [24]. This may also result in the patient seeking out multiple providers for the same illness period, which is burdensome to the healthcare system. A better approach is to listen empathetically, allowing the patient to express the emotions associated with their experience of the condition, without focusing on the truth or falsehood of the patient's claims and instead focusing on the suffering and distress that the patient has endured and continues to endure [24]. It is also important to teach self-soothing skills and monitor behavior with respect to safety. It is recommended that the clinician be curious about the patient and appreciate the role that the delusion serves in the individual's life [25]. How does a particular delusion either interfere with or facilitate everyday function? Which relationships are affected [25]? Unfortunately, though, for many doctors outside of psychiatry and psychology, there is often limited time for this best practice. In these instances, it is important to use the therapeutic alliance to motivate the patient to engage in psychotherapy and for the ophthalmologist to continue seeing the patient at regular intervals, so that the patient does not feel abandoned.

Developing a therapeutic alliance is key in helping patients to understand the value of open communication to alleviate some of the stress associated with the condition, and such a therapeutic alliance might serve as a springboard in suggesting that the patient seek help from a psychotherapist (i.e. “someone to talk to”) [24]. Aside from intensive psychotherapy, such as CBT [20, 26], a number of treatments have been proposed and used in clinical practice, including pharmacological interventions [16–20]. As a psychotic spectrum disorder, treatment may involve the use of first and second-generation antipsychotics, which in some cases can induce remission [16, 18, 20]. Initiation of antipsychotic treatment is near impossible without a therapeutic alliance, as patients typically lack insight into the psychological origins of their delusions and believe that they truly are experiencing a cutaneous condition [4, 16]. To overcome this hurdle, physicians can emphasize the additional benefits of the medication, such as its analgesic and antipruritic effects [16]. Close follow-up by psychiatry is essential in these cases.

This study stands apart from previous research on MD by underscoring the absence of identifiable lesions despite thorough ophthalmic and orbital examinations, while also illuminating the pivotal role of the patient's initial traumatic experience of colliding with a trumpet plant in precipitating the onset of the delusional disorder. While this case report offers valuable insights into the ophthalmic manifestations of MD, it also presents limitations. The focus on an individual case may limit the generalizability of findings. While the report describes observed ocular symptoms, establishing causality between MD and these manifestations remains challenging. Potential bias in reporting, along with confounding variables like concurrent medical conditions such as floppy eyelid syndrome, could influence result interpretations. Moreover, the limited follow-up period from June 2019 to February 2023 and ethical considerations regarding patient privacy and consent may restrict the depth of insight provided. For future studies, it is recommended to conduct larger scale studies to validate the findings of this case report and enhance generalizability. Longitudinal studies with extended follow-up periods would provide valuable insights into the progression and treatment outcomes of MD. Further research is needed to explore the psychological and physiological mechanisms underlying the association between traumatic experiences and the onset or exacerbation of delusional disorders in the context of MD.

Despite the lack of a clear etiology or diagnostic criteria, many patients with MD report that the disease exacts a significant toll on their quality of life, including social and occupational impairment, financial burden, and reduced physical and mental functioning. Early recognition of a delusional disorder such as Morgellons is critical in establishing a therapeutic alliance and helping the patient to reach the appropriate mental health professional(s). It also decreases burden on the healthcare system by limiting the number of clinicians seen for the same illness episode. Increased awareness is a prerequisite to identifying effective treatments and management strategies for this complex condition.

## Data Availability

No underlying data were collected or produced in this study.
